# “Iron triangle” of regulating the uterine microecology: Endometrial microbiota, immunity and endometrium

**DOI:** 10.3389/fimmu.2022.928475

**Published:** 2022-08-09

**Authors:** Na Zhu, Xuyan Yang, Qiao Liu, Yahui Chen, Xiaolan Wang, Huanhuan Li, Hong Gao

**Affiliations:** ^1^ Department of Nursing, The Second Affiliated Hospital, Hengyang Medical School, University of South China, Hengyang, China; ^2^ School of Nursing, University of South China, Hengyang, China; ^3^ Center for Reproductive Medicine, The First Affiliated Hospital, Hengyang Medical School, University of South China, Hengyang, China; ^4^ Department of Gynecology, The Second Affiliated Hospital, Hengyang Medical School, University of South China, Hengyang, China

**Keywords:** endometrial microbiota, endometrium, immunity, uterine microecology, female reproductive tract

## Abstract

The uterus is the core place for breeding new life. The balance and imbalance of uterine microecology can directly affect or even dominate the female reproductive health. Emerging data demonstrate that endometrial microbiota, endometrium and immunity play an irreplaceable role in regulating uterine microecology, forming a dynamic iron triangle relationship. Up to nowadays, it remains unclear how the three factors affect and interact with each other, which is also a frontier topic in the emerging field of reproductive tract microecology. From this new perspective, we aim to clarify the relationship and mechanism of the interaction of these three factors, especially their pairwise interactions. Finally, the limitations and future perspectives of the current studies are summarized. In general, these three factors have a dynamic relationship of mutual dependence, promotion and restriction under the physiological or pathological conditions of uterus, among which the regulatory mechanism of microbiota and immunity plays a role of bridge. These findings can provide new insights and measures for the regulation of uterine microecology, the prevention and treatment of endometrial diseases, and the further multi-disciplinary integration between microbiology, immunology and reproductive medicine.

## 1 Introduction

The uterus is a unique organ of the female upper reproductive tract, which is an important area of the human microecosystem. The balance of uterine microecology is a prerequisite for maintaining women’s reproductive health and giving birth. Throughout studies of uterine microecology, it was found that the interdependent relationship between endometrial microbiota, immunity and endometrium maintained the dynamic balance of uterine microecology (1–3). Whether the uterus is in a physiological or pathological state, uterine microbiota, immunity and endometrium can affect and interact with each other.

**Figure 1:**
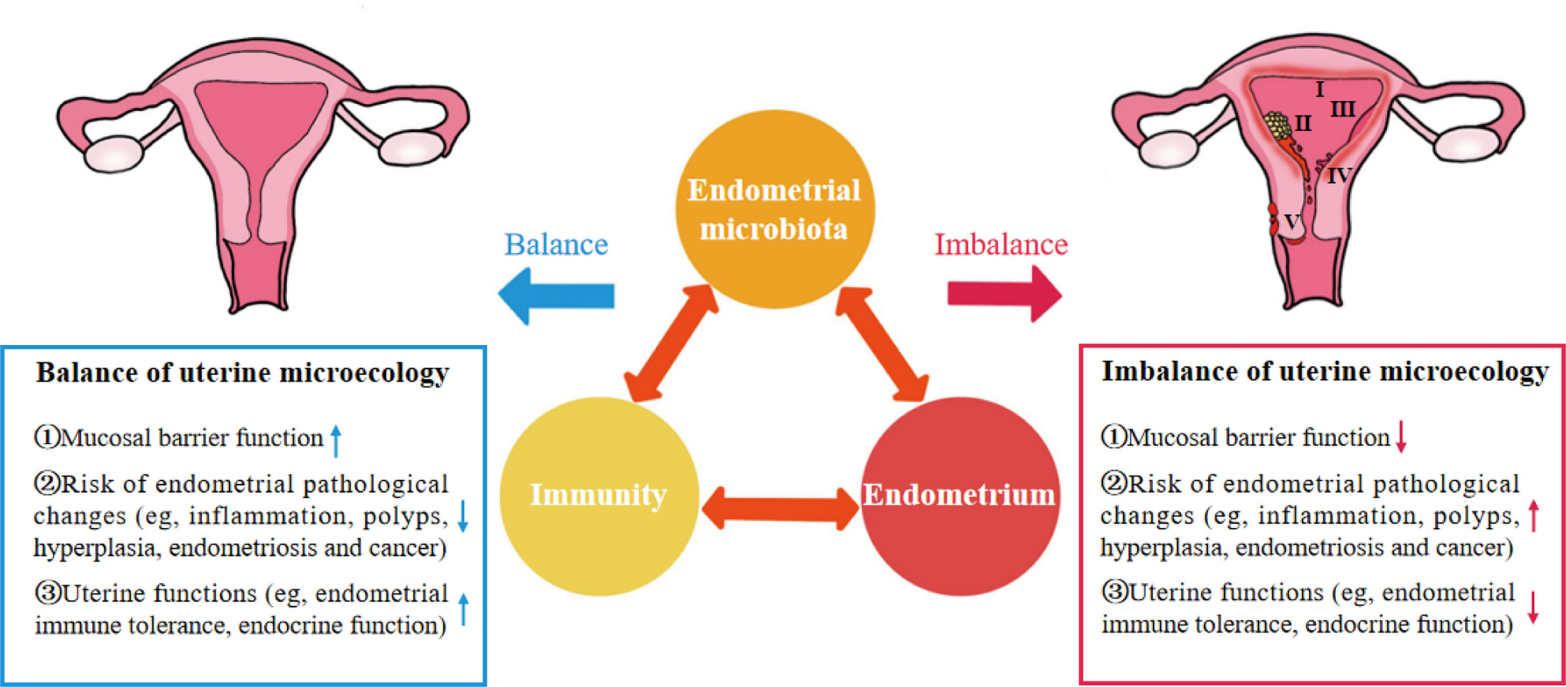
“Iron triangle” of regulating the uterine microecology: endometrial microbiota, immunity and endometrium. **I**, Chronic Endometritis; **II**, Endometrial cancer; **III**, Endometrial hyperplasia; **IV**, Endometrial polyps; **V**, Endometriosis.

As an important organ that breeds new life, the uterus was considered a sterile environment for nearly a century until the existence of bacteria was found by culturing the endometrial microbiota of healthy women in 1985 (4). With the development of gene sequencing technology, the unique, low abundance and diversity of endometrial microbiota have been gradually confirmed. The microbiota plays an important role in maintaining reproductive health or promoting the occurrence and development of endometrial disease. In a balanced uterine microecology, the endometrium is a suitable site for the growth and colonization of microbiota and provides a safe ecological niche for symbiotic bacteria through the mucosal immune system. Symbiotic bacteria not only compete for niches with pathogenic bacteria to protect the endometrium from infection but can also be sensed by immune cells to promote the development, maturation and functional perfection of immune cells. Immunoactive cells can produce cytokines to kill and remove pathogenic bacteria, enhance the barrier role of the endometrium, and promote endometrial repair and angiogenesis (1, 5, 6). There is interaction among endometrial microbiota, immunity and endometrium, and the abnormality of one of the three would cause continuous changes of the others, resulting in the imbalance of uterine microecology and bring the dysfunction of endometrium function and a series of pathological changes (**Figure 1**) (7, 8).

These findings indicate that endometrial microbiota, immunity and endometrium are important factors affecting the uterine microecology and form a dynamic iron triangle relationship in regulating uterine microecology (**Figure 1**). However, the potential mechanism of the interaction between endometrial microbiota, immunity and endometrium in the complex dynamic changes of uterine microecology remains unclear.

**Figure 2:**
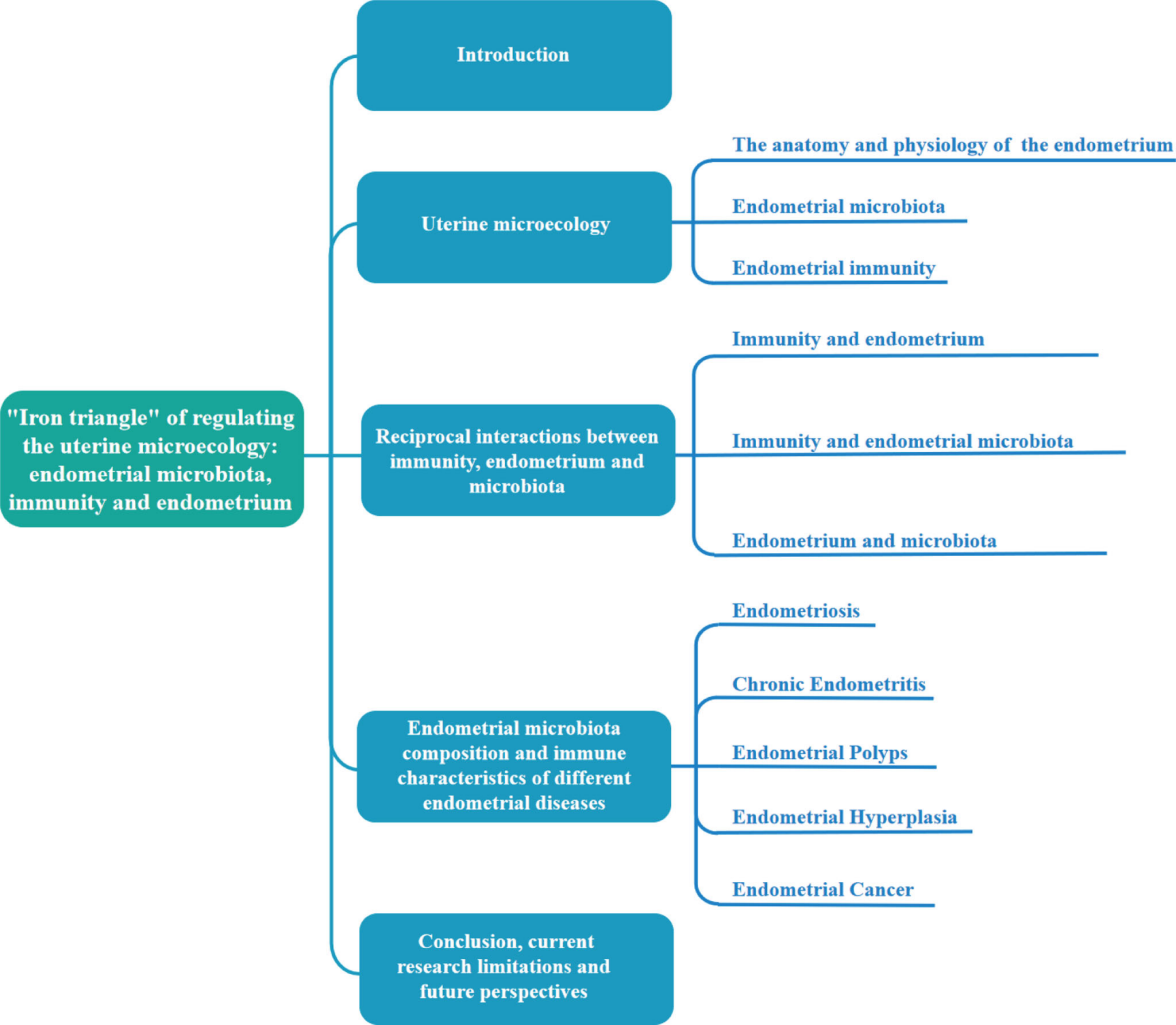
Review structure diagram.

To reveal the potential relationship and mechanism among the three factors, first, we provide some brief background of the uterine microecology, including the anatomy and physiology of endometrium, endometrial microbiota and immunity; second, we focus on the potential mechanism among endometrial microbiota, immunity and endometrium, especially their pairwise interactions; finally, we also analyzed the microbiota composition and immune characteristics in different endometrial pathological changes. The content composition of this paper is shown in **Figure 2**. We aim to clarify their roles and potential mechanisms in the balance and imbalance of microecology. This is an important aspect that cannot be ignored in regulating uterine microecology and exploring new prevention and treatment methods of reproductive diseases.

## 2 Uterine microecology

### 2.1 The anatomy and physiology of the endometrium

Normally, the uterus is a cavity muscular organ located in the centre of the pelvic cavity that looks like an inverted pear. The wider part above the uterus is called the uterine body, and the narrower part below is called the cervix. The upper end of the uterine body is the uterine fundus, and the two sides of the uterine fundus are uterine horns (9). The uterine body is composed of endometrium, myometrium and serosal from inside to outside. According to the physiological structure of the endometrium, it can be divided into two layers: the functional layer and basal layer. Two-thirds of the endometrium is the dense layer and the spongy layer, which are called the functional layer. The basal layer accounts for one-third of the endometrium near the myometrium.

The endometrium is a specific area for successful implantation, placenta formation, foetal growth and prenatal survival (10). Another unique feature of the endometrium is that hyperplasia and exfoliation occur with menstrual cycle changes. Among them, estradiol and progesterone make important contributions to the cyclical changes of endometrium. Under the effect of estradiol, the endometrial epidermis, glands, stroma and blood vessels (spiral arteries) show proliferative changes. At the secretory stage (luteal phase), the endometrium continues to thicken, the glands grow and bend more, the stroma becomes more loose and edematous, the spiral arterioles further grow and curl, and the vascular lumen expands under the common effect of estrogen and progesterone. After the withdrawal of estrogen and progesterone, the endometrial functional layer disintegrates and falls off from the basal layer during the secretory period, resulting in ischemia necrosis and denudation of the distal vascular wall and tissue, forming menstruation (1, 2).

### 2.2 Endometrial microbiota

Microecology refers to the sum of all microorganisms existing in a specific environment and their genetic materials and functions, including the interactions between microorganisms and between microorganisms and other species in the environment (11). In the past century, due to the limitations of microbial detection technology and the innate defense function of cervical mucus, the consensus was that the female uterine environment was sterile until next-generation sequencing (NGS) technology detected the type and number of normal microbiota in the endometrium, thus overturning the “sterile uterus” hypothesis (4). It has been shown that the normal microbiota can coexist with the uterus and play a leading role in uterine microecology (12–15).

Regarding the source of endometrial microbiota, scholars suggest that it may mainly come from the following pathways: ① microbes enter the blood from the intestine, oral cavity or other ways and then transported to the endometrium (blood circulation); ② ascension of microbes through the cervix; ③ retrograde transmission of microbes through fallopian tubes; ④ insertion of intrauterine device; ⑤ microbes in the lower genital tract or external environment are carried into the uterine cavity by sperm (spread with sperm); and ⑥ gynaecological procedures related to assisted reproductive technology (16–20). In addition, there may be other routes.

Compared with the composition of vaginal and cervical microbiota, but the endometrial microbial community is more unique, complex and diverse (21, 22), which may be related to the endometrial abundant blood flow, pH value, temperature and humidity, or other environmental conditions. At the same time, it is suggested that the endometrial microbiota is not a complete continuation of the vaginal and cervical microbiota. Most studies tend to divide endometrial microbiota into *Lactobacillus* dominant bacteria (>90% *Lactobacillus*) and non *Lactobacillus* dominant bacteria (<90% *Lactobacillus*, >10% other bacteria) (23). At the phylum level, the endometrial microbiota included *Firmicutes*, *Bacteroidetes*, *Proteobacteria*, and *Actinobacteria*; at the family level, *Comamonadaceae* and other (4.92%), *Tissierellaceae* and other (2.12%), *Erysipelotrichaceae* and other (1.6%); at the genus level, *Lactobacillus* (30.6%), *Pseudomonas* (9.09%), *Acinetobacter* (9.07%), *Vagococcus* (7.29%), *Sphingobium* (5%), *Arthrobacter* (3.89%), *Dysgonomonas* (3.72%), *Shewanella* (3.38%), *Pseudomonadaceae* and other (2.87%), *Delftia* (2.41%), *Sphingomonas* (1.96%), *Erysipelothrix* (1.06%), *Anaerobic Bacillus*, *Klebsiella*, *Bacteroides*, *Clostridium*, *Flavobacterium*, and unclassified; and at the species level, *Escherichia coli* (*E.coil*), *Bacteroides fragilis*, *Acanthobacteria*, *Spirillum* and *Klebsiella* (8, 24–31). To date, the composition of the endometrial microbiota has been controversial, but the genus *Lactobacillus* is a consistent discovery. It should be noted that compared with the vagina and cervix where *Lactobacillus* is dominant, the relative abundance of *Lactobacillus* in the endometrium is low, and it is usually replaced or coexisted by other bacteria.

The unique functions of the endometrial microbiota mainly include ① participating in the proliferation and apoptosis of endometrial cells; ② preventing pathogenic microorganisms from attaching to the endometrial surface and proliferating, thereby enhancing the anti-infection ability of the endometrium; and ③ binding of microbial ligands to host receptors, producing inflammatory cytokines, chemokines and antibacterial substances to participate in the regulation of the uterine immune response (1, 32). Endometrial microbiota plays an important role in embryo implantation and pregnancy maintenance. The composition of the endometrial microbiota can predict pregnancy outcomes. A high abundance of *Lactobacillus* in endometrium is conducive to better reproductive outcomes (7, 33). The imbalance of endometrial microbiota (*Streptococci*, *Staphylococci*, *Prevotella*, *E. coli*, *K. pneumoniae*, etc) is related to adverse reproductive outcomes such as repeated implantation failure (RIF), repeated pregnancy loss (RPL), biochemical pregnancy (BP) or clinical miscarriage (CM) (23, 24, 34). In addition, the disorder of endometrial microbiota plays a key role in endometrial pathological changes such as endometriosis, endometritis, polyps, hyperplasia and cancer (35–37).

### 2.3 Endometrial immunity

**Figure 3:**
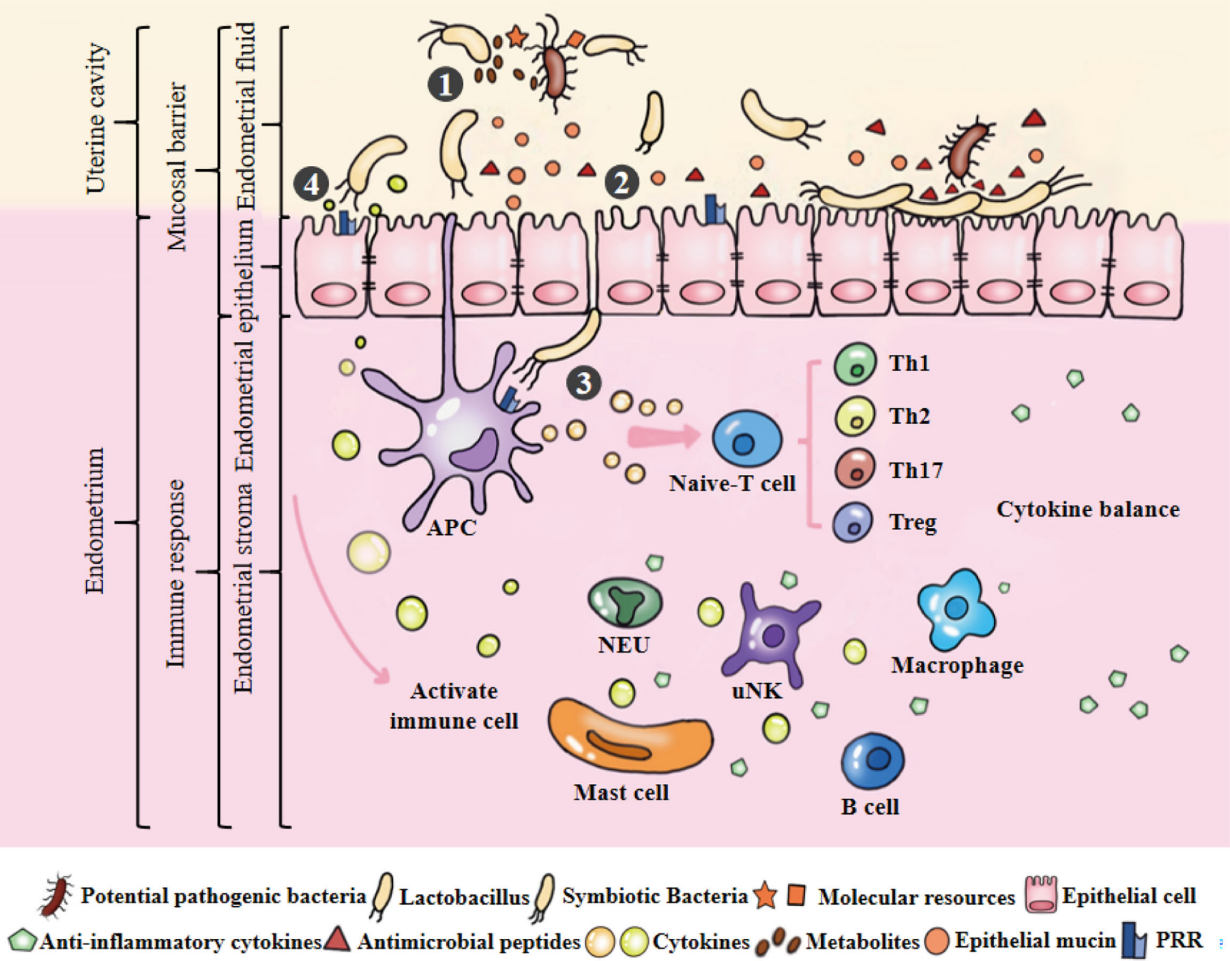
Microecological balance of the uterus. ①Symbiotic bacteria compete with pathogenic bacteria for molecular resources and occupy the uterine niche. They can also produce metabolites to kill bacteria. Among them, *Lactobacillus* plays an active role in maintaining the balance of endometrial microbiota. ②Endometrial monolayer columnar epithelial cells are closely connected to resist pathogenic bacteria, and produce natural antimicrobial peptides and mucins to strengthen this physical barrier under the stimulation of epithelial cells or symbiotic bacteria. ③APCs can sense microbiota and initiate the signal cascade by combining their receptors with the PAMPs of symbiotic bacteria to induce the development, maturation, activation, proliferation and differentiation of immune cells. ④Under the stimulation of model receptor, the production of cytokines may potentially coordinate the function and interaction of immune cells. Cytokines produced by immune cells can maintain a dynamic balance and involved in maintaining physiological functions of the uterus, such as defense against pathogenic bacteria, endometrial angiogenesis, endometrial repair and maternal-fetal immune tolerance.

Compared with mucosal parts such as the intestine and bronchus, the endometrium does not have a typical mucosal immune system. However, a large number of immune cells are distributed in the endometrium, mainly innate immune cells [macrophages (Mφs), neutrophils (NEUs), dendritic cells (DCs), uterine natural killer (uNK) cells, mast cells (MCs)] and adaptive immune cells (T cells and B cells) (38). They are widely distributed in lymphoid aggregates of the basalis and between stromal cells and epithelial cells (39). During the proliferation and secretory stage of the menstrual cycle, endometrial immune cells gradually mature to maintain the physiological immune microenvironment of the uterus and play an important role in endometrial remodelling, decidualization and embryo implantation (**Figure 3**) (1, 38). Immune cells have different functions, and their number, type and activation state are highly dependent on the hormonal environment (38, 39). In terms of quantity, immune cells in early pregnancy may reach 30~40% of the total number of uterine cells. uNK cells are the most abundant decidual immune cells (accounting for 70% of the total number of local immune cells), but DCs, Mφs, NEUs and MCs also exist. In terms of function, uNK cells can regulate the invasion of trophoblasts and enhance vascular remodelling through extravillous trophoblasts, Mφs and DCs, and their subtypes protect against infection; regulatory T cells (Tregs) can promote maternal-foetal immune tolerance (39).

Similar to other parts of reproductive tract, the endometrium may often be exposed to external microorganisms and alloantigens. Therefore, it has dual characteristics in immune potential functions (1). On the one hand, it has the function of preventing the invasion of foreign microorganisms (the activity of immune killing); on the other hand, it must have the immune tolerance or protective immunosuppression functions of receiving allogeneic antigen (embryo implantation and development). Immunoactive cells play a crucial role in the elimination of pathogenic microorganisms and promotion of immune tolerance. Considering the dynamic participation of the endometrial immune niche in endometrial function, pregnancy establishment and semi allogeneic fetal tolerance, abnormalities in this niche can lead to serious adverse pregnancy outcomes (39).

## 3 Reciprocal interactions between immunity, endometrium and microbiota

**Figure 4:**
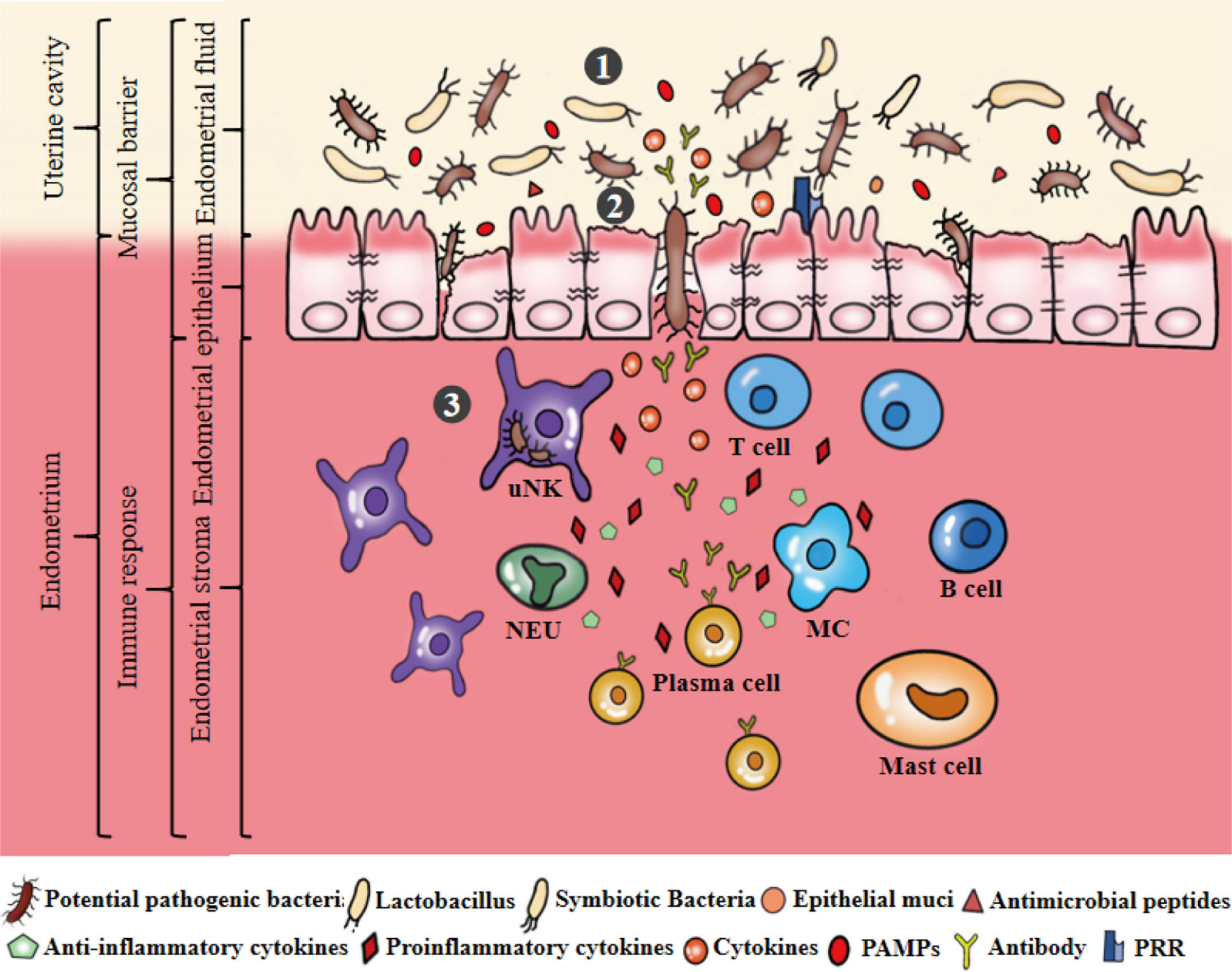
Microecological disorders of the uterus. ①The ecological imbalance of endometrial microbiota, such as the decrease of symbiotic bacteria, the increase of pathogenic bacteria. Dysregulation of bacterial metabolites, such as the abundance of PAMPs significantly greater than AMPs and mucins secretion. ②Microecological imbalance promotes pathogenic bacteria to invade, colonize and multiply on the endometrium by destroying the endometrial epithelial barrier. ③Pathogenic bacteria stimulates epithelial cells to produce a large number of inflammatory cytokines that can stimulate, recruit and aggregate immunoactive cells to produce cytokines, antibodies and other substances to eliminate and resist pathogenic bacteria. Secretion of pro-inflammatory cytokines can promote the development of endometrial inflammation.

The endometrium provides a suitable area for the microbiota. Endometrial microbiota and mucosal immunity jointly maintain the dynamic balance of endometrial physiological activities (**Figure 3**). However, the endometrial microbiota is easily affected by host characteristics, lifestyle, environmental factors and so on, eventually causing an imbalance in the endometrial microbiota. Pathogenic bacteria invade and colonize the endometrium, leading to the weakening or disappearance of uterine mucosal barrier function and the activation of an abnormal immune response, thus promoting pathological changes in the endometrium (**Figure 4**). It is worth noting that the potential mechanism and relationship between endometrial microbiota, immunity and endometrium remain to be further clarified, which is the cornerstone of research on changing female reproductive outcomes and preventing or treating uterine related diseases. Their pairwise interactions are described below.

### 3.1 Immunity and endometrium

Mucosal immunity has the function of protecting the body from pathogenic bacteria and maintaining mucosal homeostasis (40). Even though endometrium does not have a typical mucosal immune system, the interaction of immune cells, cytokines and hormones is an important part of regulating endometrial immunity and maintaining mucosal homeostasis (41). The types and efficacy of immune cells in endometrium and immune characteristics under sex hormones are described below.

#### 3.1.1 Uterine natural killer cells

uNK cells play a dominant role in the normal human endometrium. The main phenotype of uNK cells is CD3^-^CD56^+^CD16^-^, which is different from NK cells (CD3^-^CD56^dim^CD16^+^ NK cells) in peripheral blood. uNK cells and Tregs are extremely important in decidual angiogenesis, trophoblast migration and immune tolerance during pregnancy (42). In early gestation, uNK cells account for 60%~90% of decidual immune cells, which become the main immune agent in the maternal-fetal interface and then decrease in middle and late gestation (43). During normal pregnancy, uNK cells target the extracellular matrix and smooth muscle cells of the uterine spiral artery to regulate vascular remodelling and promote the delivery of nutrition and oxygen (44). NK cells are an important source of immunomodulatory cytokines and chemokines, which play an important regulatory role in the establishment of maternal-fetal immune tolerance and the development of the placenta and fetus. The effect of uNK cells on the uterine spiral artery may involve signal transduction of INF-γ, nitric oxide, and various angiogenic growth factors (VEGF) and extracellular matrix (ECM)-modifying enzymes (44). uNK cells are also involved in the regulation of trophoblast invasion into the uterus and the interaction between trophoblasts and the uterine spiral artery. Some activated immune cells can produce granulocyte macrophage colony stimulating factor (GM-CSF), eukemia inhibitory factor (LIF), colony stimulating factor-1 (CSF-1), tumour necrosis factor-α (TNF-α), transforming growth factor-β (TGF-β), interleukin-2 (IL2) and chemokine CXC motifligand 12 (CXCL12), which promote the migration and invasion of trophoblast cells (45). However, the mechanism of inhibition or promotion of this interaction remains to be studied (46, 47). In addition, uNK cells can not only promote local inflammatory responses by producing proinflammatory cytokines and chemokines such as IFN-γ, GM-CSF, IL-10 and IL-8, but also induce and activate Mφs and cytotoxic T cells (48). Paracrine mediators of uNK cells can also regulate the function of endometrial stromal cells, especially the accumulation of chemokines such as IL-8 and IL-15 (49, 50).

#### 3.1.2 Neutrophils and macrophages

NEUs and Mφs are rich sources of natural antibacterial proteins, including defensins and whey acid protein (WAP). During the perimenopause period, NEUs are labelled as CD11b^+^, CD66b^+^ and CD16b^+^ in the endometrium (51). The NEUs count is relatively constant throughout the menstrual cycle, but increases significantly during menstruation, possibly due to the surge in the level of the main neutrophil chemokine IL-8. During menstruation, NEUs can help destroy endometrial tissue by releasing elastase, which subsequently activates extracellular matrix metalloproteinase to promote endometrial abscission, intimal remodelling and intimal vascular repair (52). In addition, when the epithelial barrier is destroyed or attacked by pathogenic bacteria, the increase in NEUs aims to strengthen the innate immune defense of the mucosa (52). NEUs can produce IFN-γ in response to stimulation with LPS, IL-12, and TNF-α, and in turn, IFN-γ can activate Mφs (51). Mφs are located in the subepithelial stroma of the endometrium, especially around glands, and can express different specific markers, such as CD68^+^ and CD80 (approximately 20% of immune cells), in which CD68^+^ Mφs may play an important role in tissue clearance and tissue remodelling during the menstrual cycle (44, 53–55). Mφs are the first cells to recognize and phagocytize foreign substances (antigens). Bacterial LPS can stimulate Mφs to induce the production of biologically active proinflammatory IL-1β, which in turn induces endometrial epithelial cells to secrete the human beta-defensin-2 (HBD-2) to resist bacterial invasion (32). The antigen may stimulate the local immune response of the uterus through IFN-γ and regulate the transfer of IgA from tissue to the uterine cavity (56). Mφs are regulated by oestradiol and progesterone and are thought to play an important role in the induction of fertility and proinflammatory cytokines (39, 57, 58). TGF-β expressed by Mφs plays an important role in regulating endometrial immune function during artificial insemination and embryo implantation (59).

#### 3.1.3 Dendritic cells and mast cells

DCs are the most effective antigen capture and antigen presenting cells (APCs). They can degrade captured pathogens through lysosomal enzymes in phagosomes and lysosomes, and activate adaptive immune responses through antigen extraction, so they are highly involved in regulating mucosal surface immune responses (60). Uterine epithelial cells can secrete soluble mediators from the endometrial base to the endometrial stroma, thus inducing the tolerance phenotype of the local DCs population (61). This phenotype is characterized by reduced expression of CD83 and CD86 costimulatory molecules and stimulation and sensitivity of TLR3 and TLR4 (62). Under normal circumstances, the number of DCs is affected by the menstrual cycle and embryo implantation. For example, during the menstrual cycle, the number of mature CD83^+^ DCs in the basal layer of the endometrium is relatively stable, while the density of immature CD83^+^ DCs and CD1a^+^ DCs may increase significantly in the basal layer due to the indirect regulation of steroid hormones (63). The destruction of highly coordinated DCs cyclical changes may lead to endometrial dysfunction, thus reducing fertility and promoting the occurrence and development of gynecological diseases. In addition, two phenomena have been reported for DCs and the endometrium. First, a large number of DCs were found in the diseased endometrium and surrounding blood vessels. Second, the deletion of DCs may lead to endometrial pathological changes (64, 65). The interaction between DCs and endometrium and its specific mechanism need to be further explored. Endometrial MCs also remain relatively stable during the endometrial cycle, and play an important role in biological defense mechanisms by releasing proteases such as trypsin and chymotrypsin to activate inflammation and the immune response. These findings can reflect the key functions of MCs during menstruation. Additionally, activated MCs can produce arachidonic acid products, histamine, heparin, and a variety of multipotent cytokines and growth factors, which are closely related to tissue oedema (66).

#### 3.1.4 T and B lymphocytes

T cells are mainly concentrated in the basal layer of the endometrium and scattered between the stroma and epithelial cells. T cells are a population of lymphocytes, of which 30%~45% are CD4^+^ T cells. CD4^+^ T cells are divided into Th1 cells (5%~30%), Th2 cells (5%), Tregs (5%) and Th17 cells (2%), which play a central role in the establishment and maintenance of maternal-fetal immune tolerance (67). Th1 cells can secrete IFN-γ, TNF-β and IL-2, which activate Mφs to participate in cellular immunity to resist infection, cytotoxicity and delayed hypersensitivity caused by intracellular pathogens (68). At the same time, Th1 cells can produce TNF-α to promote inflammation. Therefore, Th1 cells are considered to be potential contributors to pathological changes in pregnancy and a major threat to fetal survival (69). Th2 cells can secrete IL-4, IL-5, IL-6, IL-10 and IL-13, which are mainly involved in humoral immunity and resistance to extracellular pathogen infection (68). Th1 and Th2 cells can inhibit each other. Th2 cells produce IL-10, which inhibits the development of Th1 cells by acting on APCs, while IFN-γ produced by Th1 cells can prevent Th2 cells from being activated (70). Th1 cells are involved in embryo transfer rejection, and Th2 cells are involved in immune tolerance during pregnancy. The increased Th1/Th2 ratio can lead to disordered estrogen and progesterone secretion, which can result in the failure of periodic cycling in the endometrium and a reduction in endometrial receptivity (71). The occurrence of this imbalance may be caused by cytotoxic factors secreted by uNK cells under the stimulation of some pathological factors. However, FoxP3^+^ Tregs can effectively inhibit the production of IFN-γ and TNF-α in the pregnant uterus, which is essential for promoting immune tolerance (72). Th17 cells can secrete the proinflammatory cytokine IL-17. Both Th17 cells and Tregs are differentiated from CD4^+^ T cells in a concentration-dependent manner under the action of TGF-β. Studies have shown that different concentrations of TGF-β have different induction effects on Th17 cell and Tregs (73). High levels of TGF-β can promote Tregs differentiation, while low levels of TGF-β can induce Th17 cell differentiation (74). CD3^+^ T cells are distributed in the basal lymphoid tissues, stroma and epithelial sites of the endometrium, accounting for only 12% of the total lymphoid cells (53, 54). Compared with peripheral blood CD3^+^ T cells, endometrial CD3^+^ T cells were composed of a higher proportion of CD8^+^ T cells (66%) and a smaller proportion of CD4^+^ T cells (33%). Among them, endometrial CD8^+^ T cells can maintain cytolysis activity during the proliferation stage, but this activity was weakened during the secretion stage (54). In early pregnancy, CD8^+^ T cells affected by hormones can reduce the expression level of cytotoxic molecules, thus maintaining immune tolerance to fetal antigens and preventing infection (75). Other studies have shown that cytokines such as IL-8 and IFN-γ produced by CD8^+^ T cells in the decidua may promote the invasion of EVT (76). Under normal circumstances, B cells are rare in the endometrium, accounting for less than 1% of the total number of immune cells, and are mainly located in the basal layer of the endometrium. B cells can secrete a variety of cytokines to participate in immune regulation and can also differentiate into plasma cells stimulated by antigens to produce a large number of antibodys-mediated immune responses. Cytokines produced by B cells, such as IL-6, IL-10, GM-CSF and IL-17, can contribute to the persistence of chronic inflammatory diseases (77).

#### 3.1.5 Hormonal regulation of the endometrial immune system

Animal studies have found that sex hormones (for example, oestradiol) can partially affect reproductive tract mucosal immunity through the regulation of polymeric immunoglobulin receptor (pIgR) mRNA expression, indicating that hormones play a role in genital tract mucosal immunity (78). The endometrial immune system is unique compared to that of other parts of the body because it must adapt to the menstrual cycle (38). During endometrial proliferation, T cells are the dominant immune cells, followed by uNK cells and Mφs. At the late secretion stage, uNK cells increase to approximately 40% of the total stromal cells and 70~80% of the total white blood cells, Mφs account for approximately 30%, and T cells decrease to less than 10% (42). In the menstruation stage, the growth of Mφs mainly promotes the shedding of endometrial tissues, accounting for 15% of the total number of white blood cells, NEUs accounts for 6~15% of the total number of cells, and the number of eosinophilic granulocytes can be as high as 5% of endometrial immune cells (39, 53, 54, 79). There are fewer uNK cells during menstruation and proliferation, which may be related to the decrease in progesterone promoting the apoptosis of uNK cells (1). Endometrial stromal cells can strongly express oestrogen receptors (ERs) and progesterone receptors (PRs) and release chemokines (42). The stimulation of progesterone and 17β-oestradiol can enhance the release of chemokines, and this effect may affect the migration and aggregation of peripheral uNK cells (80). Endometrial stromal cells of pregnant women released higher levels of chemerin than those of menopausal or nonpregnant women, suggesting that chemokines contribute to uNK cell accumulation and vascular remodelling in early pregnancy. In addition, animal experiments showed that the antigen presentation effect of uterine epithelium was the strongest in proestrus. During oestrus, the antigen presentation effect is the weakest, which is conducive to sperm entering the uterus (81).

The interaction of endometrial immune cells, cytokines and sex hormones can cause a series of changes, including the secretion of immunoglobulin antibody, the expression of protein genes related to cell proliferation and apoptosis, and the expression of leukocyte subsets in the menstrual cycle. Endometrial immunity is very special and complex, and its immune stability is crucial to the normal growth and development of maternal uterus and fetus. More studies are urgently needed to clarify the immune changes of endometrium, and to find new immune markers as diagnostic tools or predictors of endometrial disease prognosis and pregnancy outcome.

### 3.2 Immunity and endometrial microbiota

The immune system is composed of different parts, which sense the existence of microbiota and influence the composition and function of the microbiota by exerting the immune function, and play an important role in resisting the invasion of pathogenic bacteria. Similarly, some microbiota colonizing the endometrium can also affect the performance of immune function through its own or metabolites. Toll-like receptors (TLRs), antimicrobial peptides (AMPs), complement system, lipopolysaccharides (LPS), bacterial DNA, proteins or other components are important participants in the interaction between the endometrial microbiota and immunity (82). The influence of these major components of the immune system on the microbiota and the involvement of the endometrial microbiota in immune regulation are described below.

#### 3.2.1 Toll-like receptors

The defense of the endometrium against microbial infection depends largely on innate immunity. When innate immunity is activated, it leads to an acute inflammatory response, including secretion of cytokines and chemokines, recruitment of NEUs and Mφs, and phagocytosis of microorganisms and damaged cells. TLRs, NOD-like receptors (NLRs), RIC-I-like receptors (RLRs), and C-type lectin receptors (CLRs) exist on DCs, Mφs, NEUs and other innate immune cells or are expressed by epithelial cells. They have the ability to distinguish potentially pathogenic microbial components from harmless antigens. TLRs are the first line of defence against pathogenic bacterial invasion and play a key role in regulating inflammation and immune cell. Pathogen-associated molecular patterns (PAMPs) include LPS from *Gram-negative bacteria*, and flagellin, teichoteic acid, peptidoglycan, glucan, porin, mannan, bacterial RNA and DNA from *Gram-positive bacteria* (83). Each TLR is specific to a different pathogen product. For example, TLR1, TLR2 and TLR6 can recognize the lipids of *Gram-positive bacteria*, such as lipoteichoic acid; TLR4 can recognize LPS as the cell wall component of *Gram-negative bacteria*; and TLR5 can recognize flagellin. TLRs can activate mitogen-activated protein kinase (MAPK) and nuclear factor-kappa B (NF-κB) signaling pathways, regulate transcription and expression of multiple target genes such as proinflammatory cytokines, chemokines, adhesion molecules and their receptors, trigger the production of cytokines and chemokines, and upregulate costimulatory molecules on antigen-presenting cells to activate T cells and start innate immunity (84, 85). Molecules such as CD14 and LPS-binding protein (LBP) can help TLRs recognize bacteria and bacterial products, thus contributing to the activation of TLRs. Endometrial symbiotic bacteria can induce anti-inflammatory responses. For example, *Lactobacillus* can inhibit TLRs from producing pro-inflammatory cytokines and chemokines by producing lactic acid (86).

#### 3.2.2 Antimicrobial peptides

There are direct interactions between antimicrobial peptides (AMPs) and microbiota. When pattern recognition receptors (PRR) are activated, AMPs such as human neutrophil peptides, human b-defensins, secretory leucocyte proteinase inhibitor (SLPI) and elastin inhibitor (elafin) can participate in the regulation of mucosal surface inflammation through a nonspecific manner and play a role in the prevention of endometrial infection (87). Human defensins are divided into human β-defensins and human α-defensins, which are mediators of monocyte, T cells and DCs recruitment (88). The WAP motif protein family includes SLPI, trappin-2/elafin, EPPin and HE4. Both SLPI and elafin are expressed by epithelial cells and leukocytes, showing anti-protease activity and antibacterial activity. Jin et al. demonstrated that SLPI can inhibit LPS-activated NF-κB and reduce TNF-α/nitric oxide (NO) synthesis, thereby inhibiting inflammatory responses (89). The expression levels of AMPs are affected by the proinflammatory environment, elafin and sex hormones. Under the influence of these factors, the definite mechanism of their antibacterial effect is still poorly understood (90).

#### 3.2.3 Complement system

Complement is an important part of the innate immune system and is crucial for defending against microbial infections. The complement system is activated only when complement factors come into contact with antigen-antibody complexes, foreign bodies, damaged tissues, or pathogens (91). The key step for activating the complement system by recognizing non self-molecules is to activate the central C3 component through the classical pathway (antibody mediated), lectin pathway or alternative pathway (91). C3 is also present in the epithelial cells of the endometrium (92). Activation of C3 on the cell surface can stimulate the formation of membrane attack complexes on NEUs and target cells, resulting in cell damage and cell lysis, but other host cells are protected by membrane binding regulatory molecules such as CD46, CD55 and CD59 (93). Inappropriate activation of complement is involved in endometrial inflammatory injury. The differential expression of complement regulatory proteins is related to bacterial infection and cancer.

#### 3.2.4 Immune regulation of endometrial microbiota

Symbiotic bacteria can provide immune protection for the host by regulating, stimulating and regulating immune response. *Lactobacillus* is the most representative symbiotic bacteria in the reproductive tract in healthy women of childbearing age. It has regulatory effects on both nonspecific and specific immunity in the human body, including enhancing the barrier effect of mucosal immunity, improving phagocytosis by phagocytes and stimulating immune cells to produce cytokines and antibodies (IL-2, IFN, TNF-α, etc.), and inducing Th1 type cellular immune response (94). Moreover, lactic acid can induce the secretion of the anti-inflammatory cytokine IL-10, reduce the production of pro-inflammatory cytokin IL-12 in DCs, and decrease the cytotoxicity of NK cells (82).

Endometrial microbiota can exhibit a number of important metabolic pathways in an environment with high Th1 abundance, including reduction of the tricarboxylic acid (TCA) cycle, degradation of purine nucleobase, biosynthesis of L-aspartic acid and L-asparagine, and biosynthesis of thiamine diphosphate. *Phyllobacterium* and *Sphingomonas* may regulate Th1/Th2 transformation of immune cells by interfering with fat metabolism and/or carbohydrate metabolism in the endometrium (95). In the interaction of immune regulation, *Phyllobacterium* and *Sphingomonas* were positively correlated with DCs, NK cells, iTregs and B cells, but negatively correlated with Mφs (95).

It is also worth noting that some specific bacteria in the reproductive tract can inhibit immune response and promote the survival of pathogenic bacteria in cells to maintain long-term infection through multiple immune escape pathways (8). *Chlamydia trachomatis* produces a plasmid-encoded Pgp3 that neutralizes the anti-chlamydia activity of the human antimicrobial peptide LL-37 by binding to proteins to form a stable complex, which may delay the onset of a full-blown inflammatory response. Some studies have found that *Chlamydia* in the reproductive tract can block LL-37-stimulated IL-6/8 production and LL-37-induced neutrophil chemotaxis in human endometrial epithelial cells by producing Pgp3, thus promoting the survival of *Chlamydia* in infected hosts and the transmission of *Chlamydia* to new hosts. *Chlamydia* not only can use Pgp3 to improve its own adaptation the in reproductive tract epithelium, but also may reduce endometrial immune tolerance by activating myelocyte-mediated inflammation (96). Other studies have shown that *Mycobacterium tuberculosis* Rv1768 can regulate NF-κB-TNF-α signaling pathways and arachidonic acid metabolism *via* S100A9 to promote the survival of *mycobacteria* in Mφs. The immune escape of some specific bacteria is a great potential risk of adverse reproductive outcomes. In the future, it is still necessary to further identify the bacterial toxins related to bacterial escape, which will help to provide new drug targets (97).

#### 3.2.5 Endometrial proteins

Endometrial proteins play an important role in the change in endometrial microbiota. Nucleosome-binding oligomerization domain (NOD) proteins are PRRs that exist in epithelial cells, monocytes and DCs. NOD1/NOD2 protein is located in endometrial epithelium, stroma and endothelial cells. It plays a role in uterine innate immune protection and regulates menstrual inflammation. When NOD2 protein is activated by microbial peptidoglycan, it can activate the NF-κB and MAPK signalings pathways to directly produce cytokines (such as TNF and IL-1β), induce autophagy and intracellular vesicle transport, regenerate epithelial cells, and produce AMPs, thus affecting the microbial composition. The abnormal expression or activation of the NOD1/NOD2 downstream signalling pathway may increase susceptibility to uterine infection, resulting in endometrial pathological changes, reproductive tract diseases and adverse reproductive outcomes, such as abnormal menstrual bleeding, infertility, and abortion. Glycoprotein 340 (Gp340) is a kind of innate immune receptor that has a definite role in mucosal tissues. Some studies have found that the effects of Gp340 could be beneficial or harmful, depending on its conformation. For example, Gp340 can inhibit microbial infection in body fluids and promote microbial infection on mucosal surfaces (98). Gp340 also exists in genital epithelium. However, current studies mainly focus on the role of Gp340 expressed in vaginal and cervical epithelium, and the role of Gp340 in endometrium remains unclear. Moreover, IgA is expressed in mucosal tissues and inhibits the colonization and spread of pathogenic bacteria (99). Endometrial local lymphoid cells can secrete IgA (sIgA), which can protect against bacterial and viral infection. The decline in endometrial sIgA levels indicates a decrease in maternal and/or fetal innate immune function and the aggravation of endometrial microecological imbalance (100). In the special environment of the uterus, the IgA level of the endometrium can be periodically changed by hormone regulation and reach a high level in the secretory phase, but the reason is not clear. These microbial studies provide new insights into the relationship between the endometrial microbiota and mucosal immunity. For example, how do endometrial cells, cytokines and secretory antibodies jointly maintain the ecological niche balance of endometrial microbiota? How do different components of the immune system affect the abundance and species of microbiota at different endometrial stages?

The complex interactions between host innate immune systems, adaptive immune systems, microbiota, metabolites and proteins play a pivotal role in maintaining the balance of uterine microecology. At present, it is still necessary to make a further study on the relationship between endometrial microbial community and immune cells, such as exploring the commensal or pathogenic relationship of the endometrial microbiota with immune cells and possible signalling pathways, whether some bacterial communities use common mechanisms to regulate the innate immune system, and how the immune characteristics of the endometrium affect the microbial changes during embryo implantation.

### 3.3 Endometrium and microbiota

Symbiotic bacteria (normal microflora, indigenous microbiota) exist on the body surface covered by epithelial cells and are exposed to the external environment (respiratory tract, gastrointestinal tract, reproductive tract, skin, etc.) (101). Similar to the intestinal immune barrier, the endometrium can also provide a safe place for symbiotic bacterial colonization according to the growth characteristics of the normal microbiota (**Figure 3**). Studies have reported that the total amount of bacteria colonized in the uterine cavity is 10^2^ to 10^4^ times lower than the total bacterial load in the vagina (9, 102). In this unique environment, endometrium microbiota and endometrium can regulate and interact with each other, shaping the uterine pathophysiological changes.

#### 3.3.1 Endometrial regulation of the endometrial microbiota

The homeostasis of endometrial microbiota may be regulated in the following three different ways: ① the simple columnar epithelial cells of uterus can proliferate to form adenosine cells during the secretory phase of the menstrual cycle, which closely connect to form a strong anatomical barrier, hindering the exposure of resident bacteria to the uterine immune system; ② endometrial surface and fluid contain immune mediators, such as infection control molecules (AMPs, ect.), which can prevent pathogenic bacteria from coming into direct contact with epithelial cells, and have bactericidal effects on gram-negative and gram-positive bacteria, such as *E.coil* and *Staphylococcus aureus*; and ③ epithelial cells PRR and lymphocytes in the mucosal layer can resist the invasion of pathogenic bacteria (39, 103, 104).

#### 3.3.2 Hormonal regulation of the endometrial microbiota

Hormonal changes in the menstrual cycle can affect the composition of the endometrial microbiota. Oestrogen and progesterone are positively correlated with the stability of the microbial community structure, which is prone to changes during the menstrual cycle and return to its original state after the menstrual cycle (2). Most women can experience uterine microbiota changes during menstruation, including the transformation from a microbiota dominated by *L-crispatus* to microbiota dominated by *L-iners*, *G-vaginalis*, *Gram-positive cocci* or other dysbacteriotic (105). Another study reported that, *Sphingobium sp.*, *Propionibacterium* and *Carnobacterium* sp. were enriched during the proliferation period; *Propionibacterium*, *Sphingobium sp.*, *Comanonadaceae* and *Carnobacterium* sp. increased significantly during the secretion period. There is a significant difference in endometrial microbial abundance between the secretory phase and proliferative phase (106). The reason may be that there are metabolic effects between the endometrium and microbiota, especially in the pathways of prostaglandin biosynthesis and L-tryptophan metabolism. In addition, the endometrial microbiota of dysmenorrhea women is significantly different from that of healthy women. Among them, the increase in *Acinetobacter* and *Facultative anaerobic bacteria* was the most obvious (107). Nevertheless, the changes of microbiota during menstrual cycle and the mechanism of microbiota on menstruation remain to be further studied.

#### 3.3.3 Pathological effects of endometrial microbiota on endometrium

The colonization and growth of endometrial microbiota have pathophysiological effects on endometrium: ① endometrial potential pathogenic microbiota can directly or indirectly destroy the integrity of epithelial barrier; ② the metabolites secreted by endometrial microbiota and the inflammation caused by the activation of endometrial epithelial/immune cells TLR can inhibit or promote the growth of some specific bacteria; ③ the genomic stability of endometrial epithelium may be affected by regulated transcription factors of microbiota and/or epigenetic changes and/or other genomic; and ④ the increase in the number of endometrial pathogenic microbiota combined with the imbalance of genes, proteins, cytokines, hormones and other factors can make endometrium in hyperplasia, or atrophy, or hyperaemia or other states (5, 6, 20, 103).

Endometrial microbiota plays an important role in the occurrence and development of endometrial diseases. Microbiota such as *Lactobacillus*, *E.coil*, *Streptococcus*, *Gardnerella* and *Pseudomonas* are common in endometrial lesions. The composition of the microbiota varies with the pathological state of the endometrium. In endometrial polyps, *Lactobacillus*, *Streptococcus*, *Gardnerella*, *Bifidobacterium*, *Alteromonas* and *Archaea* were increased and *Sphingomonas*, *Enterobacter* and *Pseudomonas* were decreased (26). *Firmicutes* and *Proteus* can lead to local endometrial hyperplasia by affecting the level of oestrogen in blood. The microbial composition of endometrial cancer is significantly different from that of benign tumours. The detectable microbiota includes *Atopobium*, *Trichomonas Acinetobacter*, *Pseudomonas*, *Clostridium*, *Porphyrinomonas* and *E.coil.* The changes in endometrial microbiota can also be associated with mucosal infection and inflammation, such as decidualitis, chorioamnitis and other obstetric diseases (108). The presence of specific pathogenic bacteria may also activate antiangiogenic pathways, leading to changes in trophoblast and endothelial function, thereby inducing preeclampsia (109). Importantly, the composition of the endometrial microbiota can also predict reproductive outcomes. When the proportion of endometrial microbiota such as *Klebsiella pneumoniae*, *Staphylococcus*, *Enterococcus*, *E.coil* and *Streptococcus* is relatively high, the incidence of adverse reproductive outcomes is higher (110, 111). These studies indicate that regulating the microbiota of the endometrium can improve reproductive diseases or reproductive outcomes.

As mentioned above, the endometrium may modulate the evolution, composition, diversity, and homeostasis of the microbiota through various pathways, such as mucosal barriers, hormones and other means. Disorders of the endometrial microbiota contributes to pathological development in the endometrium. Their complex relationships can focus on exploring how microbiota colonize and survive in the endometrium and how to create a potentially favourable microenvironment for embryo implantation. More importantly, regulating the microbiota of the endometrium may become a new strategy for the prevention and treatment of infertility and female reproductive tract diseases. Future studies should focus on the functional changes of the endometrial microbiome and its pathogenesis in different diseases.

## 4 Endometrial microbial composition and immune characteristics of different endometrial diseases

The interaction between endometrial microbiota, endometrium and immune system is one of the important mechanisms of the occurrence and development of endometrial diseases. By consulting the relevant literature, it can be found that there are differences in microbial composition and immune response (including immune cells and inflammatory cytokines) in different endometrial diseases, such as endometriosis, chronic endometritis, endometrial polyps and endometrial hyperplasia (**Table 1**). This may indicate that the underlying mechanisms of different endometrial diseases mediated by the endometrial microbiota and immune system are different. Therefore, endometrial microbial composition and immune characteristics in different endometrial diseases deserve further discussion, and are expected to become targets for the prevention and treatment of various endometrial diseases.

**Table 1:** Endometrial microbiota composition and immune response in different endometrial disease.

Endometrial Diseases	Endometrial microbiota	Immune cell	Inflammatory factor
Endometriosis	*Lactobacillus* **↓** (112), *Staphylococcus* **↑** (30), *Gardnerella* **↑** (113), *Streptococcus* **↑** (30, 113), *Enterococcus* **↑** (30, 112, 113), *Moraxellaceae* **↑** (30), *Alishewanella* **↑** (112), *Prevotella* **↑** (114), *Acinetobacter* **↑** (115),* Vagococcus* **↑** (115), *Comamonas* **↑** (115), *Escherichia coli* **↑** (113), *Pseudomonas* **↑** (112, 115)	Mφs**↑** (39, 116–118), iDC**↑** (39, 119, 120), mDC**↓** (39, 119, 120), uNK**↓** (39, 121), NEUs**↑** (119), Tregs**↑** (39, 122), MCs**↑** (39, 120), CD8^+^ T cells**↓** (123)	IL-4**↑** (124, 125), IL-10**↑** (125–127), TGF-β**↑** (126–128), IL-1**↑** (116), IL-6**↑** (127, 129, 130), IL-8**↑** (123, 129, 131–133), IL-4**↑** (134), TNF-α**↑** (117, 132, 135), IFN-γ**↑** (126, 133), IL-13**↓** (136), IL-12**↑** (136), IL-1β**↑** (137)
Chronic endometritis	*Proteobacteria* **↓** (26), *Gardnerella* **↑** (138, 139), *Neisseria* **↑** (140), *Dialister* **↑** (139), *Bifidobacterium* **↑** (139), *Enterobacteriaceae* **↑** (140), *Enterococcus* **↑** (141, 142), *Streptococcus* **↑** (140, 142), *Klebsiella pneumoniae* **↑** (140), *Prevotella* **↑** (139), *Phyllobacterium* **↑** (95), *Sphingomonas* **↑** (95), *Anaerococcus* **↑** (139), *Actinobacteria* **↑** (139), *Staphylococcus* **↑** (140, 142), *Acinetobacter* **↑** (139)	B cells**↑** (95, 143–145), uNK**↑** (95), uNK**↓** (143, 145), Th1**↑** (95, 144), Th2**↓** (144, 146), Tregs**↓** (146, 147), M_2_ **↑** (146), Th17**↑** (95), Mφs**↑** (147), mDC**↑** (147), CD8^+^ T cells**↑** (147)	TGF-β**↓** (143, 146), TGF-β**↑** (148), IL-10**↓** (143, 146), IL-17**↑** (143, 146), IL-11**↓** (143, 145), TNF-α**↑** (144, 145, 148, 149), IFN-γ**↑** (144, 145, 148), IL-6**↑** (144, 149), IL-4**↓** (144), IL-5**↓** (144), IL-13**↓** (144), IL-12**↑** (148), IL-1**↑** (149)
Endometrial polyps	*Lactobacillus* **↑** (26), *Bacteroides* **↑** (26, 150), *Proteobacteria* **↓** (26, 150), *Pseudomonas* **↓** (26, 150) *Enterococcus faecalis* **↑** (150), *Staphylococcus aureus* **↑** (150), *Staphylococcus epidermidis* **↑ (** 150), *Bifidobacterium* **↑** (26), *Gardnerella* **↑** (26), *Streptococcus* **↑** (26), *Alteromonas* **↑** (26), *Prevotella* **↑** (26), *Escherichia coli* **↑** (26)	MCs**↑** (151), Tregs**↑** (151), Tregs**↓** (152), Th17**↑** (152, 153), γδ T cells**↑** (154), Mφs**↑** (153)	TNF**↑** (153), TNF-α**↓** (155), TGF-β**↓** (152), TGF-β_1_ **↑** (153), IL-17**↑** (152, 153), IL-1β**↑** (153), IL-6**↑** (153), IL-23**↑** (153), IFN-γ**↑** (152, 156, 157)
Endometrial hyperplasia	*Lactobacillus* **↓** (158), *Acinetobacter* **↓** (158), *Klebsiella* **↓** (158), *Firmicute* **↑** (158, 159), *Proteobacteria* **↑** (158, 159), *Actinobacteria* **↑** (158, 159), *Fusobacteria* **↑** (150, 158), *Bacteroides* **↑** (158, 159), *Escherichia coli* **↑** (150, 158), *Bacteroides fragilis* **↑** (158)	simple EH: CD45^+^ T cells**↑** (160), NEUs**↑** (161), Mφs**↑** (161), complex EH: CD45^+^ T cells**↓** (161), NEUs**↓** (161), Mφs**↑** (161), uNK**↓** (161), Tregs**↑** (160), CD8 +T cells**↑** (160)	TNF-α**↓** (162–164), TNF-α**↑** (12, 164, 165), simplex and complex hyperplasia: TNF-α**↑** (164), atypical hyperplasia: TNF-α**↓** (164), TGF-β**↑** (166–168), IL-1β**↑** (165, 169), IL-6**↑** (165)
Endometrial cancer	*Lactobacillus* **↓** (158, 170)*, Prevotella* **↑** (158, 170, 171), *Klebsiella* **↑** (158, 171), *Muribaculum* **↑** (170), *Pelomonas* **↑** (170, 171), *Nocardioides* **↑** (170), *Anaerostipes* **↑** (172), *ph2* **↑** (172), *Treponema* **↑** (172), *Atopobium* **↑** (172), *Bacteroides* **↑** (158, 171, 172), *Arthrospira* **↑** (172), *Dialister*↑ (172), *Peptoniphilus*↑ (172), *1-68*↑ (172), *Ruminococcus*↑ (172), *Porphyromonas*↑ (172), *Anaerotruncus*↑ (172) *Bacteroides fragilis*↑ (158), *Pseudomonas uter*↑ (158)	M_2_ **↑** (173–176), CD8^+^ T cells**↓** (175–179), Tregs**↑** (175, 176, 178, 180), uNK**↓** (175, 176, 178, 179), DCs**↓** (175, 176, 178), CD4 ^+^ T cells**↑** (178), B cells**↑** (175), NEUs**↑** (176), Th17**↑** (180), Tc17**↑** (180)	IL-6**↑** (175, 181), IL-17**↑** (180), IL-17A**↑** (175), IL-10**↑** (175, 176, 180), IFN-γ**↓** (175, 177), IL-8**↑** (182, 183), TGF-β**↑** (175, 176, 178, 184), TNF-α**↓** (175), IL-1β**↑** (176)

### 4.1 Endometriosis

**Figure 5:**
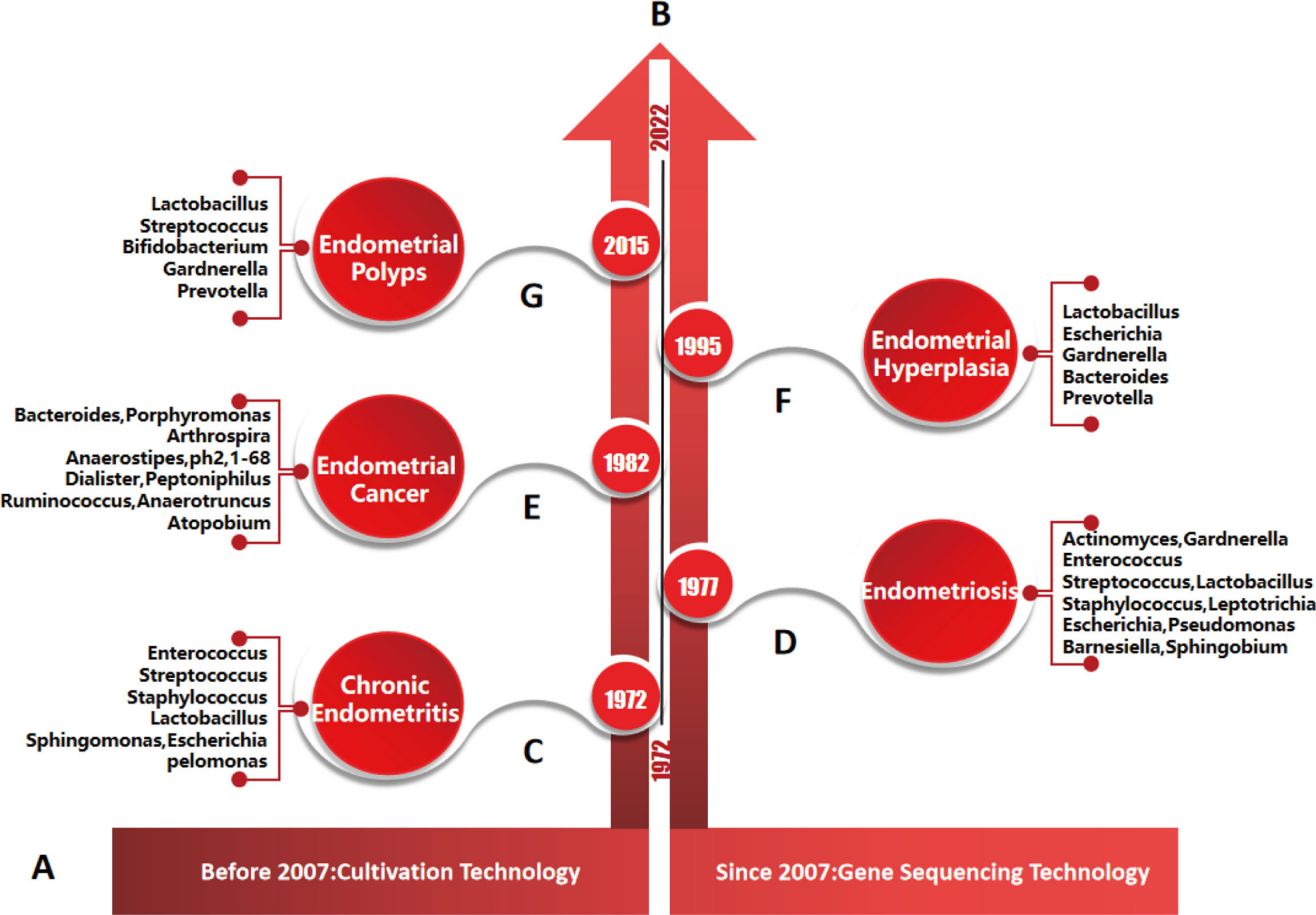
Timeline of endometrial diseases, major microorganisms and their detection methods. **(A)**, detection methods of endometrial microorganisms. According to the literature, culture technology was mainly used before 2007, and gene sequencing technology was mainly used after 2007. **(B)**, research progress of endometrial diseases and microorganisms: studies on the relationships between, chronic endometritis, endometriosis, endometrial cancer, endometrial hyperplasia, endometrial polyps and microorganisms were first reported in 1972, 1977, 1982, 1995 and 2015, respectively. **(C–G)**, main species of endometrial microbiota were detected by genus sequencing technology in different endometrial diseases.

Endometriosis (EMs) is mainly characterized by the growth of endometrial tissues (glands and stroma) outside the uterine cavity (the endometrium muscular layer, ovaries, pelvis, other body sites around genital organs or away from the genital organs) (185, 186). EMs is a common and frequent disease that is associated with infertility. Statistically, 10~15% of women of childbearing age suffer from it (187). The aetiology of EMs is complex, and disturbance of human microecology is an important factor. Research on the endometriosis microbiota first began in 1977, and subsequently a large number of studies have been reported (**Figure 5**) (188). Khanl et al. reported that the endometrial microbiota of EMs patients was mainly *Staphylococcus*, *Gardnerella*, *Streptococcus* and *Enterococcus*, followed by *Actinomycetes*, *Corynebacterium*, *Clostridium*, *Prevotella* and *Propionibacterium* (30). The different stages of EMs have different microbiota compositions. In stage I EMs, the abundance ratio of *Lactobacillus*, *Clostridium* and *Campylobacter* was higher, while the abundance of *Gardnerella* was lower. In stage II EMs, the proportion of *E.coli/Shigella*, *Megalococcus* and *Gastrostreptococcus* was higher. After the destruction of the endometrial basal layer caused the endometrium to invade the myometrium, the reduction or increase of endometrial many microbiota also increased the diversity of the microbial community (the depletion or enrichment of *Sphingobium* sp., *Pseudomonas viridislava*, *methylophilaceae* and other bacteria). However, there are few studies on endometrial microbiota of adenomyosis, and more prospective population cohorts are urgently needed for verification (8). At the same time, there is no clear evidence to support the mechanism of differences in bacterial abundance in endometriosis or adenomyosis.

Endometrial microbiota participate in the occurrence and development of EMs by affecting immune function (185). Immune cells (especially Mφs, immature DCs, Tregs, B cells and NK cells) seem to be a key factor in the development of EMs (39). In initiating the host defense mechanism, the activation of immune cells releases a large number of cytokines, prostaglandins and complement components, including VEGF, TNF-α, monocyte chemoattractant protein-1 (MCP-1), TGF-β, IL-6, IL-17, and IL-22 (39, 189). In this process, the abnormal expression of the TLRs signalling pathway plays a mediating role. Endotoxin/LPS produced by bacteria such as *E.coil* can promote the development of EMs by activating the TLR-4 pathway (190). On the other hand, it can also stimulate abdominal cavity Mφs to produce a large number of pro-inflammatory immune factors (IL-6, IL-8 and TNF-α), stimulating the growth of abnormal endometrium in a dose-dependent manner. Moreover, even though EMs is a benign disease, it has the characteristics of malignant tumours, such as tissue invasion, local dissemination, recurrence and metastasis, which may involve the mutation of proto oncogenes and tumour suppressor genes (191).

EMs seriously affect women’s health and quality of life. Endometrial microbiota and immune response promote the occurrence and development of EMs, but there are still some unsolved problems: ① most studies focus on the relationship between microbiota and the aetiology, symptom formation and malignant transformation of EMs, but fail to understand its specific internal mechanism, and ② the distribution characteristics and predictive role of endometrial microbiota in different stages or lesion sites of EMs need to be further discussed. These are helpful to explore some characteristic bacteria or abundance proportions in the microbial community to provide an important reference for the diagnostic markers of EMs.

### 4.2 Chronic endometritis

Chronic endometritis (CE) is a condition involving the breakdown of the peaceful coexistence balance between the microbiota and the host immune system in the endometrium. As early as 1972, it was reported that the imbalance of endometrial microbiota could lead to the occurrence and development of endometritis (**Figure 5**) (192). Common pathogens of CE include *Chlamydia trachomatis*, *Neisseria gonorrhoeae*, *Bifidobacterium*, *Staphylococcus*, *Streptococcus*, *Bacillus proteus*, *Gardnerella vaginalis*, *Enterococcus faecalis*, *Klebsiella pneumoniae*, *E.coil*, *Pseudomonas aeruginosa*, *Mycobacterium tuberculosis* (especially in developing countries), *Mycoplasma genitalium*, *Mycoplasma hominis* and *Ureaplasma urealyticum* (138, 140, 141, 193). CE caused by HIV virus, herpes simplex virus and cytomegalovirus is rare. The colonization of the microbiota in the endometrium leads to persistent inflammation of the endometrium, which is one of the main causes of infertility. However, the diagnosis of CE is not common in the clinic due to the nonspecific clinical symptoms, expensive microbial detection and the usage of preventive antibiotics, and its relationship with infertility is not recognized. There have also been different reports on the species and abundance of endometrial microbiota in CE patients because of the differences in sample size, environmental factors, sample standardized treatment and related sequencing methods, so the treatment of CE has become a clinical problem (194).

CE is an infectious disease accompanied by an abnormal immune response, which is pathologically characterized by infiltration of endometrial stromal plasmacytes (ESPCs). B cells are the progenitors of plasma cells. When pathogenic bacteria invade endometrium, B cells enter the endometrial stroma under the action of CXCL_13_, CXCL_1_ and selectin E, and differentiate into plasma cells under the stimulation of antigen. Plasma cells are competitive immune cells. The increase in their number would indicate endometrial infection, chronic inflammation or autoimmune diseases. The severity of CE is correlated with the increase in plasma cells and their antibody expression. Kitaya et al. showed that the ESPCs of CE patients highly expressed immunoglobulins, including IgM, IgA1, IgA2, IgG1 and IgG2 (195). The abnormal expression of antibodies on plasma cells was involved in the disorder of the immune microenvironment to some extent, thus causing the impairment of endometrial receptivity and the implantation failure of embryos (196). The imbalanced proportions of uNK cells, T cells, DCs and Mφs are also involved in the process of destroying endometrial receptivity (57, 62, 75, 197). The balance of Th17/Tregs is one of the most important conditions in the pregnancy process (198). Tregs can inhibit the lymphocyte reaction through direct contact or indirectly produce cytokines such as TGF-β and IL-10 to reduce the maternal rejection of allogeneic embryos and promote the successful implantation of embryos. However, the decreased expression of TGF-β and IL-10 in CE patients suggests impaired Tregs function, which may lead to endometrial inflammatory reactions, fibrosis and embryo implantation failure (146).

CE can reduce the fertility of women of childbearing age. But for a long time, CE has been ignored in clinical practice, and the interaction between the endometrial microbiota and immune system in the occurrence and development of CE has not been comprehensively and deeply discussed. Further exploration of this potential mechanism is conducive to promoting female reproductive health and improving the success rate of pregnancy.

### 4.3 Endometrial polyps

Endometrial polyps (EPs) are caused by local endometrial local hyperplasia, which can affect 7.80%~34.9% of women worldwide (199). The risk of suffering EPs increases with age. The pathogenesis of EPs is still unclear. At present, it is thought that the occurrence of EPs is closely related to the expression imbalance of local oestrogen, progesterone, hormone receptors, cytokines, etc. In 2015, researchers proposed for the first time that the endometrial microbiota was involved in the occurrence of EPs (**Figure 5**) (200). The endometrial microbiota of patients with EPs mainly includes anaerobic bacteria (*Bacteroides* is the most common) and aerobic bacteria(such as *Proteobacteria*, *Enterococcus faecalis*, *Staphylococcus aureus* and *Staphylococcus epidermidis*) (150). Compared with healthy women, the proportion of *Lactobacillus*, *Bifidobacterium*, *Gardnerella*, *Streptococcus*, *Alteromonas* and *Prevotella* was higher in EPs patients, while the proportion of *Pseudomonas*, *Enterobacter* and *Sphingomonas* was lower (26). Another study showed that the low abundance of *E.coil* in the endometrium may promote the excessive growth of endometrial tissue (26). To date, the species of pathogenic bacteria on EPs are still controversial.

EPs are a macroscopic manifestation of the inflammatory process. It is characterized by local infiltration of NK cells (CD56), Mφs (CD68), leukocytes (CD45), and plasma cells (CD138). The activity of these cells leads to excessive abnormal hyperplasia of the endometrium (201). Additionally, the increased activity of MCs may play a major role in the occurrence of EPs (103). The number of activated MCs in EPs patients was more than 7 times higher than that in the normal population. This result is consistent with El-Hamarneh et al. (151). Extensive infiltration of MCs is accompanied by recruitment of Tregs, which can increase the risk and recurrence of EPs (151, 152). Other studies have shown that the increase of *Lactobacillus* and *Bifidobacterium* in the endometrium may promote the occurrence of EPs (26). These two bacteria play an important role in promoting cell proliferation and inhibiting cell apoptosis by activating epidermal growth factor receptor (EGFR). It can also activate nicotinamide adenine dinucleotide phosphate (NADPH) oxidases (NOXs) to cataly the production of reactive oxygen species (ROS) and then promote cell migration and proliferation, resulting in local endometrial hyperplasia and polyp formation (202). EPs are directly related to the decline in fertility, but the mechanism of infertility or abortion caused by EPs is complex and uncertain. Bozkurt et al. proved that the expression levels of NF-κB1 and NF-κBp65 in EPs patients were significantly higher than those in unexplained infertility and normal fertility, while they were significantly decreased after removal of uterine polyps (203). This indicates that the increased expression of NF-κB may be one of the potential mechanisms of damaging the endometrium and reducing fertility. LPS produced by pathogenic bacteria promoted the overexpression of PD-1 and programmed death-1 ligand (PD-L1), which also plays a crucial role in the occurrence and development of EPs (195).

EPs in women of childbearing age are also a potential pathogenic mechanism of other reproductive diseases such as CE and infertility. At present, most studies focus on the treatment of EPs, but the recurrence rate is still high. Further study on the changes in microbiota, immune characteristics and pathogenesis of endometrial polyps under different pathological changes may be an effective way to reduce the risk of EPs.

### 4.4 Endometrial hyperplasia

Endometrial hyperplasia (EH) is a typical morphological change of the endometrium. It is characterized by an increased gland-to-stroma ratio in the endometrium compared with the ormal proliferative endometrium (204). The aetiology of EH may be mainly similar to endometrial cancer (EC), including the dysregulation of hypothalamic-pituitary-ovarian axis, genetics, obesity, estrogen therapy, etc. In 1995, the study of Fujita M et al. first showed that the uterine microbiota may also be involved in the occurrence and development of EH (**Figure 5**) (205). In the endometrial microbiota of EH patients, the relative abundance of *Firmicutes*, *Proteobacteria*, *Actinobacteria*, *Fusobacteria*, *Bacteroides*, *E.coil* and *Bacteroides fragilis* increased, while *Lactobacillus* decreased (158). Kubyshkin et al. also reported that the endometrium of EH patients contained a large amount of *Bacteroidetes* and *Firmicutes* and found that the oestrogen level was significantly higher than that of normal endometrium and peripheral blood (165). This may be due to the increase in local free oestrogen levels caused by the increase in β-glucuronidase activity through the colonization of *Proteobacteria* and *Firmicutes*. Hormone imbalance can only promote the transformation from normal endometrium to simple hyperplasia, while the release of inflammatory cytokines (such as IL-6, IL-1β and TNF-α) can promote the abnormal hyperplasia of endometrial glands and stroma. These findings can better explain the pathogenesis of endometrial hyperplasia caused by hormones and inflammatory signals (165).

The pathogenesis of EH is the most complicated and involves the role of the endometrial microbiota and immune cells. Some studies have found that with the development of tissue proliferation, the relative abundance of *E.coil* and *Bacteroides fragile* increased, which may be related to the increased local IL-6/Treg ratios, reflecting the involvement of endometrial microbiota in the occurrence and development of EH through inflammation and immune regulation (158). In different types of EH, such as proliferative phase (PP), simple endometrial hyperplasia (SEH) and complex endometrial hyperplasia (CEH), there were significant differences in the types and numbers of endometrial immune cell subsets (161). Compared with PP, the proportion of NEUs and CD45^+^ cells and the subtypes of MCs and T cells were significantly increased in SEH patients, and the number of NK cells was decreased. Compared with the PP and SEH groups, the subsets of NK cells, T cells and CD45^+^ cells were significantly decreased in the CEH group, but the proportion of MCs increased. Kubyshkin et al. suggested that immune inflammatory cytokines such as IL-1β, IL-6 and TNF-α may also play a vital role in the development of EH (165).

Local immune environment analysis is helpful to monitor disease progression and assess the risk of EH developing into EC. Exploring the specific role of these immune cell subsets from the perspective of microbiota may reveal a new mechanism for the occurrence and severity of EH.

### 4.5 Endometrial cancer

Endometrial cancer (EC) is the sixth most common malignancy in women, accounting for more than 2% of global female cancer deaths (206, 207). Genetic alterations and environmental factors are major risk factors for EC. Since 1982, a growing number of studies have suggested that an imbalance of genital tract microbiota and/or specific bacteria plays a positive role in the occurrence and/or progression and/or metastasis of gynaecological malignancies (**Figure 5**) (208–210).

The local microbial composition and biomass of EC patients changed significantly. Li et al. reported that the proportions of *Prevotella, Muribaculum Pelomonas and Nocardioides* in EC patients were higher than those in healthy people, while the proportion of *Oscillibacter* was lower (170). Walther-antonio et al. classified the genital tract microbiota of women with benign uterine disease and EC (172). In the conjoint analysis of uterus and lower reproductive tract samples, they found that 12 bacterial taxa were significantly enriched at the genus level in the genital tract of EC patients, which were *Anaerostipes, ph2, Treponema, Atopobium, Bacteroides, Arthrospira, Dialister, Peptoniphilus, 1-68, Ruminococcus, Porphyromonas* and *Anaerotruncus*. Further analysis showed that *Atopobium* sp. and *Porphyromonas* sp. were common in the samples of EC patients, but virtually absented in patients with benign uterine disease.

The endometrial microbiota of EC varied at different stages of pathological development. At the phylum level, the relative abundance of *Proteobacteria* was the highest in stage II EC, followed by stage IB, and the lowest in stage IA. At the genus level, there was no significant difference in the relative abundance of *Bacteroides* between stage IB and II of EC, but they were significantly higher than stage IA; the relative abundance of *Lactobacillus* was low at all stages. At the species level, the relative abundance of *E.coil* increased with the progress of EC; the relative abundance of *Bacteroides fragilis* was the highest in stage IB EC, followed by stage II, and the lowest in stage IA (158). The composition of the endometrial microbiota varied among different pathological types of EC (211). Compared with endometrial adenocarcinoma (EAC), uterine serous carcinoma (USC) has significantly less microbial diversity, dominated by *Pseudomonas uteri*. In addition, the results of Walsh et al. showed that *Porphyrinomonas* and *Anaerococcus* were enriched in the endometrium of postmenopausal EC patients (212). Other studies have shown that an uncultured representative of *Porphyromonas* sp.(99% match to *P. somerae*) and *Atopobium vaginae* are closely related to the occurrence of EC and atypical hyperplasia, especially with higher vaginal pH (>4.5) (172). This may be related to the endometrial inflammation caused by bacterial infection, but the specific mechanism is not clear.

The microbiota may stimulate inflammation and then induce immunopathological changes and eventually lead to tumours. Tumour associated macrophages (TAMs) have a high degree of plasticity and diversity, which can promote tumour cell invasion, infiltration, extravasation and sustained growth at the site of metastasis and inhibit the cytolytic T cell response (173). It was found that TAMs in the endometrium of EC patients were mainly distributed around the tumour stroma, and their number was closely related to cell differentiation, myometrial infiltration, loss of progesterone receptors and prognosis (213, 214). The frequent infiltration of lymphocytes into the tumour and peritumoral area may be related to the recurrence of EC. Kondratiev et al. reported that the number of CD8^+^ T cells in the peritumoural area >10/field is an independent prognostic factor associated with improved survival rate (215).

Human leukocyte antigen G (HLA-G) is an immunosuppressive molecule that is highly representative of pathological conditions such as malignant transformation. Ben et al. first reported the study of sHLA-G subtype expression and dimers in EC patients and verified the relationship between HLA-G molecules and EC progression (216). The number of sHLA-G in EC patients was higher than that in healthy people. The expression of HLA-G5 in EC patients was higher than that of HLA-G1. The level of HLA-G5 in stage III EC patients is higher than that in patients with stage I and II EC, reflecting the potential significance of HLA-G5 in tumour invasiveness and early clinical development (217). A study reported that 75% of EC patients expressed HLA-G monomers and only 25% expressed HLA-G dimers (218). In contrast to HLA-G dimers, HLA-G monomers can bind to all HLA-G receptors (ILT2, ILT4 and KIR2DL4), indicating that HLA-G dimers seem to have more effective immunosuppressive potential than HLA-G monomers (38, 219, 220). However, there are few studies on the effect of sHLA-G on EC.

In recent years, the role of TLRs and NOD genes in inflammatory diseases and tumours has attracted much more attention. With regard to EC, genetic mutations associated with innate immune responses have not been studied, but altered inflammatory responses may make it easy for individuals to develop EC (221). TLR9 can specifically recognize unmethylated CpG motifs containing DNA in bacteria (222). Activation of TLR9 contributes to increased transcriptional activation of NF-κB, prompting DCs to mature and release pro-inflammatory cytokines (223). The TLR9 rs5743836 polymorphism could be more active after TNF-α or LPS stimulation, enhancing TLR9 binding affinity to NF-κB and resulting in increased release of proinflammatory mediators. However, Ashton et al. found that the combination of the variant alleles for TLR9 rs5743836 and rs187084 seems to have a protective effect on the development of EC, which may be related to the effective clearance of pathogenic bacteria in endometrium (224). Therefore, the potential biological mechanism of TLR9 polymorphism in EC needs to be further explored in population-based studies (224).

Reproductive tract microbial imbalance and immune dysfunction are the driving factors of gynaecological malignant tumours. Focusing on the relationship between microbial imbalance, immune response and gene expression and its potential mechanism will be of great significance for searching for new signaling pathway, treatment means, diagnosis methods and prognostic biomarkers of EC.

According to the above discussion, it has been proven that the imbalance between endometrial microbiota, endometrium and immunity can cause the occurrence and development of a variety of endometrial diseases. There are differences in microbiota composition and immune response in different types or pathological stages of endometrial diseases. However, it is also worth noting that microbial composition and immune response have some commonalities in these endometrial diseases. First, *Lactobacillus*, *E.coil*, *Bifidobacterium*, *Bacteroides*, *Streptococcus*, *Staphylococcus*, *Pseudomonas*, *Prevotella* and *Gardnerella* are common bacteria in endometrial diseases. Compared with healthy endometria, the number of *Firmicutes* and *Lactobacillus* in endometrial diseases decreased, while the number of *Proteobacteria* (such as *Staphylococcus*, *E.coil*, etc.), *Bacteroidetes* (such as *Bacteroides fragilis*, *Prevotella*, *Bacteroides*, etc.) and *Actinobacteria* (such as *Gardnerella*, *Bifidobacteria*, etc.) increased. Second, uNK cells, DCs, Mφs, NEUs, T cells and B cells play an important role in the immune response of endometrial diseases. The levels of pro-inflammatory cytokines (such as IL-1, IL-6, and IFN-γ) increased, while the levels of anti-inflammatory cytokines increased or decreased. These findings should be considered in the clinical prevention and treatment of endometrial diseases and the improvement of reproductive outcomes.

## 5 Conclusion, current research limitations and future perspectives

The interaction between endometrial microbiota, endometrium, and immunity can shape the balance or imbalance of women’s uterine microecology. This study provides a deep understanding of the relationship between the endometrial microbiota, endometrium, and immunity from different viewpoints, which provides a valuable theoretical basis for carrying out endometrial-related and pregnancy outcome-related studies. We can draw the following conclusions. ① Endometrial microbiota, endometrium and immunity are three indispensable parts of uterine microecology. These three factors can promote and/or restrict each other to form a dynamic iron triangle relationship, shaping the balance or imbalance of uterine microecology, which is directly related to uterine health or diseases. ② Under physiological conditions, the endometrium is not only a nutrient-rich habitat for the colonization and growth of normal microbiota, but also the first physical barrier against infection. Endometrial immune cells can sense and contact the microbiota through the endometrium, thereby activating the cascade signal of immune cells, which is conducive to promoting the growth and differentiation of endometrial immune cells, the balance of endometrial microbiota and the normal exertion of endometrial function to maintain the dynamic balance of uterine microecology. ③ Under pathological conditions, the imbalance of uterine microecology is often accompanied by disorders of endometrial microbiota, damage to the endometrium and abnormal activation of the immune response. For example, when pathogenic bacteria invade the endometrium, they can cause endometrial microbiota disorder, destroy the integrity of endometrial tissue and abnormally activate the immune response.

Of note, the relationships among endometrial microbiota, endometrium, and immunity should not be ignored, and there are still many mysteries of their interaction and collaboration mechanisms remain to be explored. Current research limitations and future perspectives are shown in **Table 2**. There is still a long way to go in the endometrial microbiota, endometrium and immunity. On this basis, we will further summarize the relationships between the placenta, fetus, immunity and maternal decidual microbiota during pregnancy. With the development of each new research, we will get closer to the truth about the interaction between endometrial microbiota, endometrium and immunity in the physiological and pathological state of the uterus, which will provide us with more effective methods of diagnosis, treatment means and prevention measures, thus improving female reproductive health.

**Table 2:** Current research limitations and future perspectives.

Limitations	Reasons	Future Perspectives
1.There are few studies on the endometrial microbiota and immunity.	①Technical methods for identifying endometrial microbiota are not widely used; ②The anatomical site of the upper reproductive tract is special and difficult to sample.	①To study the endometrial microbiota composition and immunity response (including the composition, proportion and cytokine changes of immune cells, etc.) of healthy women under physiological conditions (such as different age, menstrual cycle, pregnancy, etc.).
		②Whether there is a “core microbiota” in endometrium.
		③To compare the effects of antibiotics on endometrial microbiota during different growth stages or metabolic states of microbiota.
		④To develop and study the detection technology of reproductive tract microbiota applied to various endometrial diseases and pregnancy complications.
		⑤To strengthen the continuous and systematic study of the whole reproductive tract microbiota and immune response(including vagina, cervix, uterine cavity, fallopian tube and ovary); to compare the specific composition, abundance and function of the whole reproductive tract microbiota in the menstrual cycle and different pregnancies stages, and their effects on the development and function of immune cells.
2.The research results of endometrial microbiota are different.	①There is no unified standard for detection technology and sampling method of microbiota; ②Samples are susceptible to DNA contamination from the background of sampling, extraction kits, PCR, sequencing reagents, etc.; ③The sample size was insufficient.	①Consensus on standards for microbiological testing techniques and analytical methods is urgently needed.
		②According to the analysis results of the first small sample size, the study design and verification testing process were strictly controlled to improve the accuracy of testing results.
		③To carry out the multicenter, large sample size cohort study; to consider the differences of patient groups in different backgrounds such as geographical environment, race and living habits; to combine patient cohort studies with animal studies to more accurately interpret host microbiota characteristics.
3.The mechanism between endometrial microbiota and/or immunity and/or endometrium needs further research.	①The dynamic changes among endometrial microbiota, immunity and endometrium add the complexity of the mechanism; ②Individual microecology varies greatly.	①To analyze the interaction between endometrial microbiota and host immunity. For example, how do endometrial microbiota induce immune tolerance and persist in the host body through immune regulation; whether there is a common signaling pathway in the regulation of endometrial innate or adaptive immunity by certain endometrial microbiota under physiological or pathological conditions; how the interaction of endometrial microbiota and immunity affects endometrial receptivity and embryo implantation through gene regulation.
		②By culturing and isolating strains in the uterus to intervene in endometrial cells, tissues or animals *in vitro*, especially in the presence of other pathogenic bacteria, to explore the immune pro-inflammatory response of pathogenic bacteria and the key cellular pathways induced by microbial metabolites in endometrial epithelium.
		③To detect and screen the biomarkers for early disease damage or subclinical infections and use computer technology(such as bayesian statistics, artificial neural networks) for data analysis can bring a new idea for clinical treatments of endometrial diseases and tumors——endometrial microbiota targeting method.
		④Microbiota may cause cancer by promoting inflammation, and may also affect cancer cells by releasing carcinogenic molecules(such as genotoxins) and producing metabolites. Therefore, therapeutic microecological agents are urgently needed to be developed.
		⑤The current microbial detection methods can not completely determine all pathogenic bacteria in the endometrium and pathogenic abundance of microbiota. These will require improved microbiological techniques(such as DNA macrogenome sequencing) to analyze gene function, metabolites, metabolic pathways and the relationship between microbiota and their hosts, so as to obtain more comprehensive microbial information.

## Author contributions

All authors conceived and designed the review. NZ, XY, HG, QL and YC undertook the initial research. NZ, XY, HG, YC, QL, XW and HL were involved in writing. HG reviewed the manuscript. All authors contributed to the article and approved the submitted version.

## Funding

This work was supported by Natural Science Foundation of Hunan Province,China (Grant No. 2021JJ40472),the scientific research project of Hunan Health Commission,China (Grant No. 202212034414).

## Acknowledgments

We thank American Journal Experts (https://www.aje.con) for editing this manuscript.

## Conflict of interest

The authors declare that the research was conducted in the absence of any commercial or financial relationships that could be construed as a potential conflict of interest.

## Publisher’s note

All claims expressed in this article are solely those of the authors and do not necessarily represent those of their affiliated organizations, or those of the publisher, the editors and the reviewers. Any product that may be evaluated in this article, or claim that may be made by its manufacturer, is not guaranteed or endorsed by the publisher.
